# Supplementary material for: Evaluation of Collagen Alterations in Early Precursor Lesions of High Grade Serous Ovarian Cancer by Second Harmonic Generation Microscopy and Mass Spectrometry
Source: Cancers (Basel). 2021 Jun 4;13(11):2794. doi: 10.3390/cancers13112794 (PMC8200041; doi:10.3390/cancers13112794)
Supplement: Supplementary file 1 [file cancers-13-02794-s001.zip › supp only2.pdf]

Supporting Information (SI)

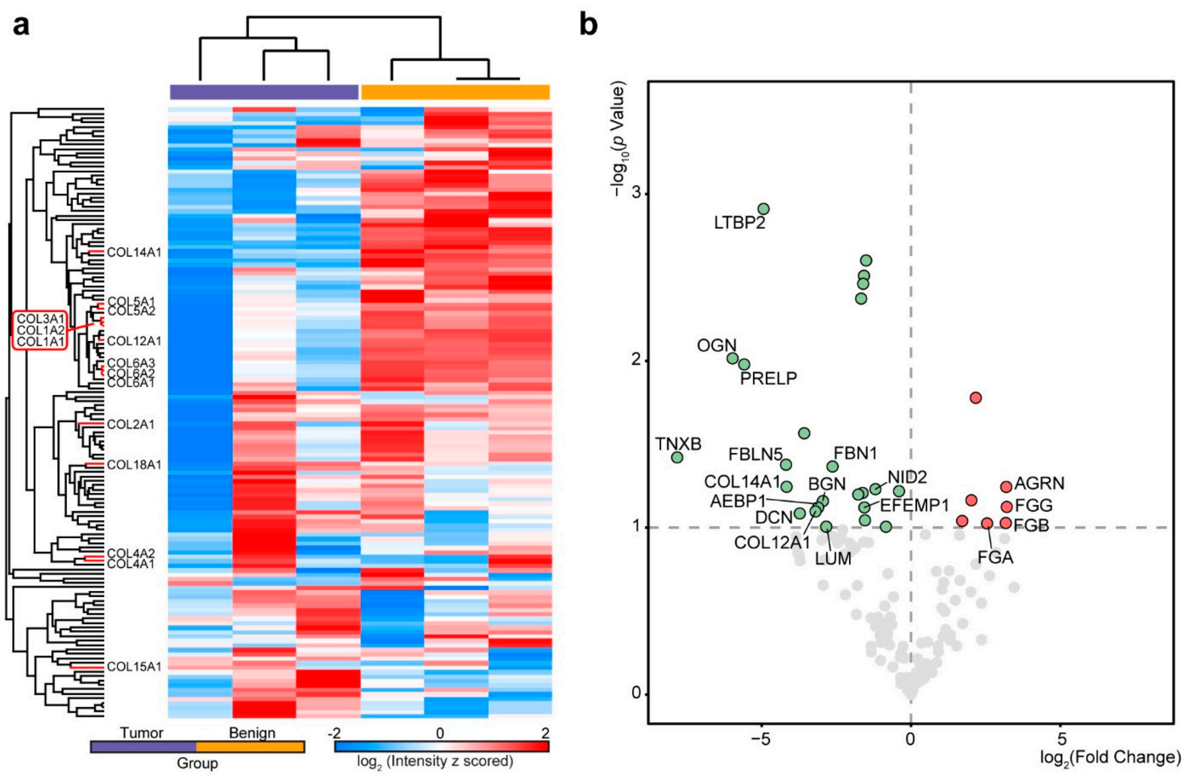

Figure S1. ECM alterations in ovarian tumor versus benign tissue revealed by mass spectrometry. **(a)** Hierarchical clustering of LFQ ion intensities of all quantified ECM proteins in different samples. Collagens are highlighted in red in row clustering and annotated with gene name. **(b)** Volcano plot showing pairwise comparison of ECM protein expression levels between tumor versus benign tissue. Points above horizontal dash lines represent significantly altered proteins (two-sided *t* test, p value < 0.1). Significantly down and up-regulated proteins are shown in green and red, respectively. Core matrisome proteins are annotated in the figure.

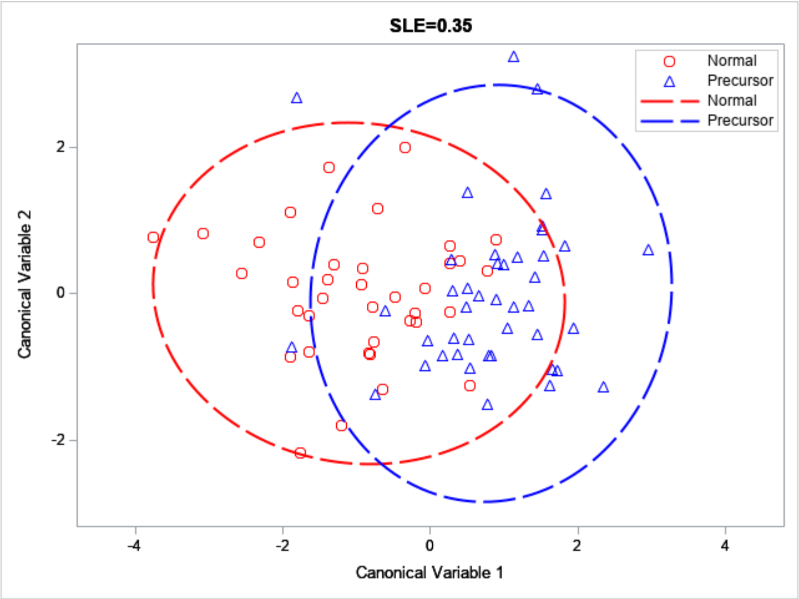

Figure S2. Scatter plot of the distal normal and precursor groups with 95% confidence ellipses depicted.

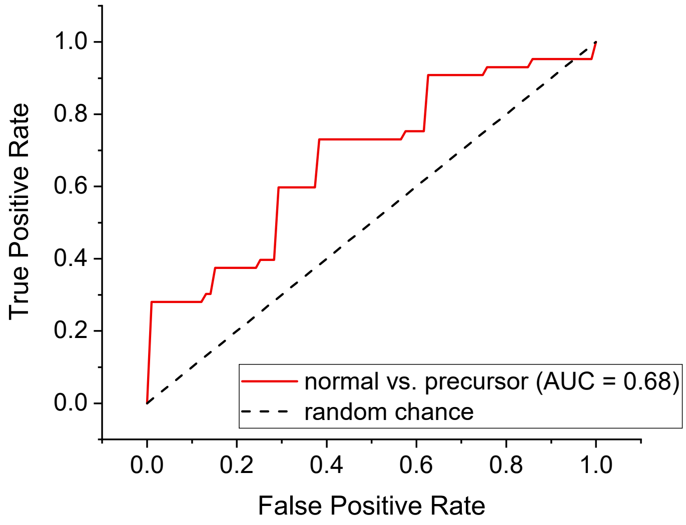

Figure S3. ROC curve and AUROC for distal normal vs. precursor classification

Sample	Patient Age	Menopausal Status	BRCA I/II Mutation Status	Cancer Present in Ovary?	Concurrent Cancer in Case?	Neoadjuvant Therapy Administered?	Chemotherapy (specific regimens)?
S1	72	Post	Unknown	No	No	No	N/A
S2	46	Post (due to previous hysterectomy)	BRCA1	No	No	No	N/A
S3	48	Post	BRCA2	Yes - small focus of serous carcinoma on surface of one ovary	Very early serous carcinoma of the fallopian tube, just next to the STIC	No	3 cycles Taxol/carboplatin x 3 weeks. Taxol 175 mg/m2, carbo AUC=6. Dose reduction of Taxol to 150 due to neuropathy
S4	52	Post	Negative	No	Concurrent endocervical carcinoma; metastatic sites of endocervical carcinoma included ovaries and one fallopian tube (not the one with STIC)	No	Platinum-based chemotherapy with radiation
S5	67	Post	Unknown	No	Concurrent uterine endometrioid carcinoma	No	N/A
S6	64	Post	Negative	No	Concurrent uterine endometrioid carcinoma	No	N/A
S7	69	Post	Unknown	No	Concurrent uterine endometrioid carcinoma	No	N/A
S8	72	Post	BRCA2	No	No	No	N/A
S9	67	Post	Unknown	No	Concurrent uterine endometrioid carcinoma	No	N/A
S10	63	Post	Negative	No	Concurrent uterine serous carcinoma, confined to uterus	No	Carboplatin and paclitaxel x 1 cycle; then switched to Taxotere/carboplatin for cycles 2-6.
S11	55	Post	BRCA1	No	No	No	N/A
S12	84	Post	Unknown	No	No	No	N/A

Supp Table 1. Summary information for all STIC tissues

Only present in normal		Only present in tumor	
Gene	Category	Gene	Category
IGFBP5	ECM Glycoproteins	ADAM10	ECM Regulators
VIT	ECM Glycoproteins	PLOD3	ECM Regulators
GPC6	ECM-affiliated Proteins	CILP	ECM Glycoproteins
PLXDC2	ECM-affiliated Proteins	FCN3	ECM-affiliated Proteins
WISP2	ECM Glycoproteins	CST3	ECM Regulators
COL8A2	Collagens	C1QA	ECM-affiliated Proteins
COL21A1	Collagens	F13B	ECM Regulators
SMOC2	ECM Glycoproteins	CTSH	ECM Regulators
SBSPON	ECM Glycoproteins	MUC1	ECM-affiliated Proteins
PODN	Proteoglycans	PZP	ECM Regulators
COL8A1	Collagens	S100A1	Secreted Factors
CILP2	ECM Glycoproteins	COL7A1	Collagens

Supp Table 2. Differential expression of ECM proteins in normal ovary and HGSOC tumor samples
